# Methyl-CpG Binding Protein 2 (Mecp2) Regulates Sensory Function Through Sema5b and Robo2

**DOI:** 10.3389/fncel.2015.00481

**Published:** 2015-12-21

**Authors:** Wan Y. Leong, Zhi H. Lim, Vladimir Korzh, Thomas Pietri, Eyleen L. K. Goh

**Affiliations:** ^1^Program in Neuroscience and Behavioral Disorder, Duke-NUS Graduate Medical School, SingaporeSingapore; ^2^Institute of Molecular and Cell Biology, SingaporeSingapore; ^3^Department of Biological Sciences, National University of Singapore, SingaporeSingapore; ^4^Institut de Biologie de l’École Normale Supérieure, Institut National de la Santé et de la Recherche Médicale U1024, Centre National de la Recherche Scientifique UMR 8197Paris, France; ^5^Department of Physiology, Yong Loo Lin School of Medicine, National University of Singapore, SingaporeSingapore; ^6^KK Research Centre, KK Women’s and Children’s Hospital, SingaporeSingapore

**Keywords:** Mecp2, trigeminal ganglion, sensory functions, Rett syndrome, neurodevelopmental disorder, axon guidance cues, Robo2, Sema5b

## Abstract

Mutations in the gene encoding the *MECP2* underlies Rett syndrome, a neurodevelopmental disorder in young females. Although reduced pain sensitivity in Rett syndrome patients and in partial MeCP2 deficient mice had been reported, these previous studies focused predominantly on motor impairments. Therefore, it is still unknown how MeCP2 is involved in these sensory defects. In addition, the human disease manifestations where males with mutations in *MECP2* gene normally do not survive and females show typical neurological symptoms only after 18 months of age, is profoundly different in MeCP2-deficient mouse where all animals survived, and males but not females displayed Rett syndrome phenotypes at an early age. Thus, the mecp2-deficient zebrafish serves as an additional animal model to aid in deciphering the role and mechanisms of Mecp2 in neurodevelopment. Here, we used two independent methods of silencing expression of Mecp2 in zebrafish to uncover a novel role of Mecp2 in trigeminal ganglion sensory neurons during the embryonic development. *mecp2*-null mutation and morpholino-mediated silencing of Mecp2 in the zebrafish embryos resulted in defects in peripheral innervation of trigeminal sensory neurons and consequently affecting the sensory function. These defects were demonstrated to be dependent on the expression of Sema5b and Robo2. The expression of both proteins together could better overcome the defects caused by Mecp2 deficiency as compared to the expression of either Sema5b or Robo2 alone. Sema5b and Robo2 were downregulated upon Mecp2 silencing or in *mecp2*-null embryos, and Chromatin immunoprecipitation (ChIP) assay using antibody against Mecp2 was able to pull down specific regions of both Sema5b and Robo2 promoters, showing interaction between Mecp2 and the promoters of both genes. In addition, cell-specific expression of Mecp2 can overcome the innervation and sensory response defects in Mecp2 morphants indicating that these MeCP2-mediated defects are cell-autonomous. The sensory deficits caused by Mecp2 deficiency mirror the diminished sensory response observed in Rett syndrome patients. This suggests that zebrafish could be an unconventional but useful model for this disorder manifesting defects that are not easily studied in full using rodent models.

## Introduction

Mutations in the gene encoding the Methyl-CpG binding protein 2 (*MECP2*) underlies Rett syndrome, a neurodevelopmental disorder presented with mental retardation, autistic behavior, compromised sensory sensations and loss of previously acquired cognitive milestones, including purposeful hand use and expressive language, in young females. Other clinical features of Rett syndrome include impairment of sleep pattern, breathing disturbance when awake, peripheral vasomotor disturbance, autonomic dysfunction (cold, blue extremities), dystonia, progressive scoliosis and diminished response to pain (Hagberg et al., 1983; Amir et al., 1999; Armstrong, 2005; Downs et al., 2010; Neul et al., 2010). It has been documented that about 90% of all Rett syndrome cases resulted from mutations in the X-linked *MECP2* (Amir et al., 1999; Shahbazian and Zoghbi, 2001; Armstrong, 2005; Bienvenu and Chelly, 2006).

MeCP2 is a multifunctional protein that was first identified by its ability to bind to methylated DNA (Lewis et al., 1992; Bird, 2008; Guy et al., 2011). Earlier studies on MeCP2 demonstrated its role as transcriptional repressor for a selected set of target genes (Nan et al., 1997; Chandler et al., 1999). Subsequent studies showed that MeCP2 may be involved in both transcriptional repression or activation, depending on the molecular context (Chahrour et al., 2008; Ben-Shachar et al., 2009; Guy et al., 2011). In addition, MeCP2 was shown to interact with the RNA-binding protein Y box-binding protein 1 and regulates the splicing of reporter minigenes, and is possibly responsible for the aberrant alternative splicing patterns in a mouse model of Rett syndrome (Young et al., 2005). Thus, mutations in *MECP2* are expected to alter expression of its downstream target genes with the consequences of impaired neuronal development and function.

Alterations in MeCP2 expression have been detected in autism spectrum disorders as well as in non-syndromic mental retardations (Chahrour and Zoghbi, 2007). MeCP2 is ubiquitously expressed, but its critical function in the mammalian brain is suggested by the abundant expression of MeCP2 in the CNS (Skene et al., 2010). Therefore, most of the current studies on Rett syndrome and MeCP2 are focused on the development of neurons in the developing or adult CNS (Ma et al., 2015; Zhao et al., 2015). Moreover, the panembryonic *Mecp2* gene knockout or the brain-specific gene knockout in mice showed similar neurological phenotypes (Chen et al., 2001; Guy et al., 2001). These studies suggested a requirement for MeCP2 in the normal development of the nervous system. The importance of MeCP2 in embryonic development as well as postnatal physiological processes underlies the majority of disease etiologies associated with Rett syndrome. Although reduced pain sensitivity in Rett syndrome patients and reduced pain recognition in partial MeCP2 deficient mice have been reported (Samaco et al., 2008; Downs et al., 2010), it is still unknown how MeCP2 is involved in these sensory responses.

Like many X-linked disorders, Rett syndrome patients displayed mosaic expression of mutant and normal MeCP2 protein in different cell types, resulting in significant variations in phenotypes and clinical severity (Shahbazian and Zoghbi, 2001; Christodoulou and Weaving, 2003; Skene et al., 2010). Moreover, not much is known with regards to the differences of the various MeCP2 protein isoforms and mutations during early development as well as in disease pathology. The *MECP2* gene is also present in non-mammalian vertebrates, including the zebrafish *Danio rerio* (Coverdale et al., 2004) and *mecp2*-null zebrafish also shows altered motor behaviors (Pietri et al., 2013). The mecp2-null zebrafish model enables the screening for genes involved in early development and will aid in deciphering the role and mechanisms of Mecp2 in vertebrate neurodevelopment. Using both *mecp2*-null mutants and *mecp2* morpholino-mediated knockdown approach, we have identified an important role for and also established underlying mechanisms of Mecp2 function in vertebrate neurodevelopment. Specifically, we found Mecp2 regulating projections of sensory neurons and sensory responses, at least in part, through directly activating transcription of specific axon guidance cues, Sema5b and Robo2.

## Results

### Mecp2 Plays a Role in Embryonic Development of Zebrafish

**FIGURE 1:**
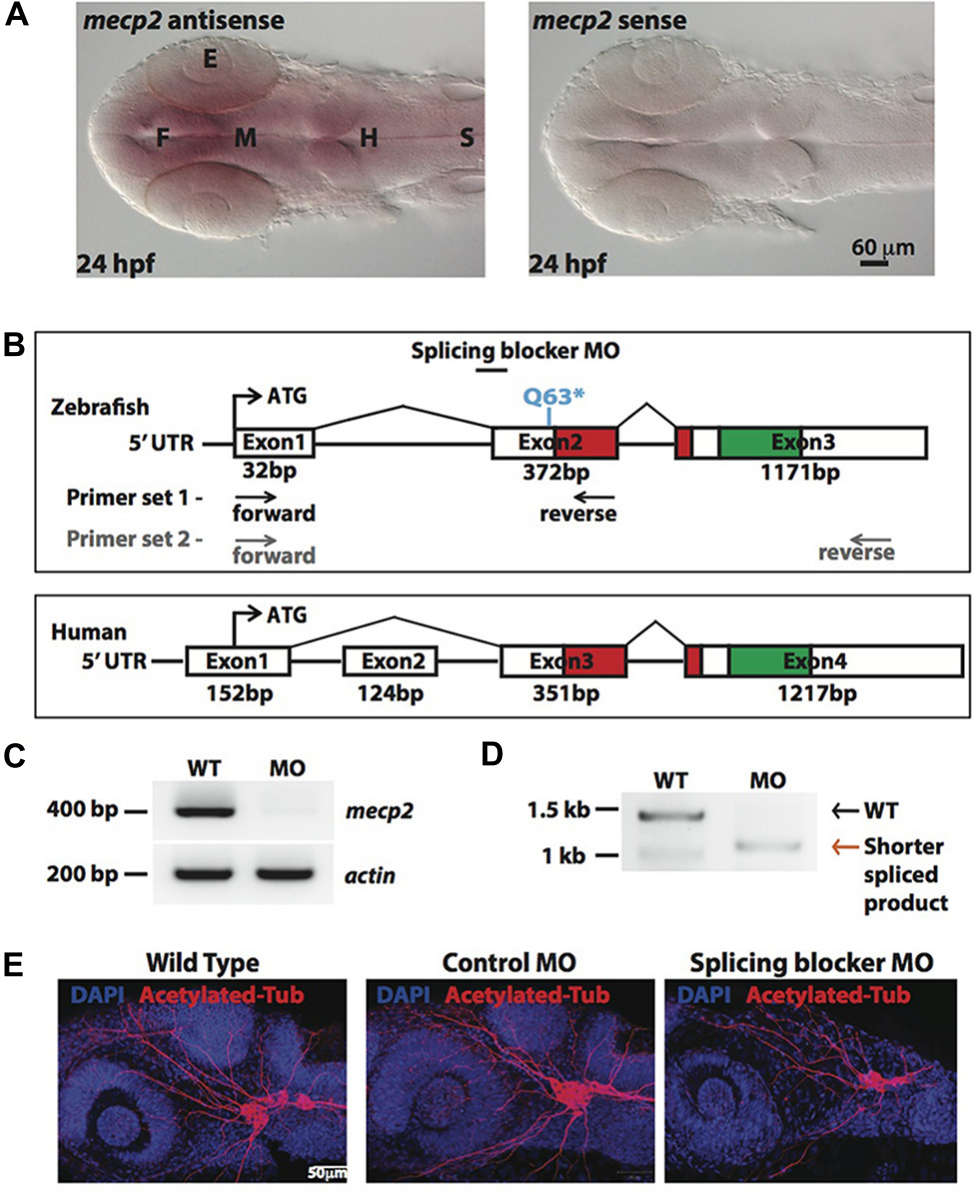
**Knocking down Mecp2 in zebrafish.** Mecp2 is ubiquitously expressed throughout the CNS at 24 hpf **(A)**. The embryos are displayed as whole mounts, dorsal view (F- forebrain, M - midbrain and H-hindbrain, S - spinal cord, E- eye) Scale bar = 60 μm. Schematic diagram shows gene structure of the zebrafish *mecp2* and the mammalian (human or mouse) MeCP2α isoform **(B)**. The zebrafish *mecp2* gene consists of 3 exons with the main domains highlighted in red (Methyl-CpG-binding domain) and green (transcriptional repressor domain). The splice blocking morpholino targets the intron 1/exon 2 boundary on *mecp2* pre-mRNA. Arrows below Zebrafish *mecp2* gene show the positions where the forward and reverse primers were targeted [primer set 1 with black arrows for experiment in **(C)** and primer set 2 with gray arrows for **(D)**]. The specificity of morpholino was determined by RT-PCR **(B,C)**. Representative gel images showing normal splicing of *mecp2* as a band of 400 bp with actin (206 bp) as the control **(C)**. Representative gel image showing normal splicing of *mecp2* in WT (1455 bp) or blocked splicing band (exon skipping) (1086 bp) in *mecp2* spliced MO injected embryos **(D)**. Representative images showing gross morphological changes in TG neurons projections induced by *mecp2* MO during development of zebrafish at 24 hpf **(E)**. Scale bar = 50 μm.

*In situ*-hybridization showed that *mecp2* in the zebrafish is ubiquitously expressed in the central nervous system (CNS), with the highest level of expression found in the forebrain (**Figure 1A**). We next compared genomic organization of mammalian (human) *MECP2* and zebrafish *mecp2* gene (**Figure 1B**). Zebrafish *mecp2* contains three exons while human *MECP2* contains four exons. Although zebrafish *mecp2* does not have exon 2 of human *MECP2*, it also contains two main domains critical for its functions. These are a methyl-cytosine binding domain (MBD) and a transcriptional repression domain (TRD) that shares 85.9 and 54% identity respectively with mammalian species (Coverdale et al., 2004). *mecp2*-null mutants (Q63^∗^) was a nonsense mutation generated through *N*-ethyl-*N*-nitrosourea (ENU)-mutagenesis. This Q63^∗^ position is located directly before the MBD domain leading to a loss of function (Pietri et al., 2013). Since there are no available antibodies against zebrafish Mecp2, we designed MO oligonucleotide corresponding to the sequence of the splice acceptor site at the intron 1-exon 2 border on zebrafish *mecp2*. This is so that the efficiency and specificity of splicing blocker MO can be determined by RT-PCR (**Figures 1C,D**). Using two sets of primers (a forward primer on exon 1 and a reverse primer on exon 2 or exon 3) (**Figure 1B**), a deletion of the 372 bp fragment in the exon 2 was detected in morphant pre-mRNA resulting in absence (primer set 1 – **Figure 1C**) or shorter PCR product (primer set 2 –**Figure 1D**). MO corresponding to the sequence of the ATG translation initiation site was also tested and the morphants showed similar phenotypes as those injected with splicing blocker MO (data not shown).

MeCP2 was previously shown to regulate neurite growth and axon targeting in the hippocampus, motor cortex and olfactory system of the mouse (Matarazzo et al., 2004; Belichenko et al., 2009; Degano et al., 2009; Palmer et al., 2012). Thus, we first examined gross axonal projections using an axon marker, acetylated tubulin (AcTub) at 24 h post fertilization (hpf). The projections of the sensory neurons of the trigeminal ganglion (TG) are among the earliest detected with anti-AcTub antibody (Metcalfe et al., 1990). Uninjected WT embryos or embryos injected with control or *mecp2* MO at one-cell stage were examined at 24 hpf for gross morphologies of their projections from TG neurons (**Figure 1D**). Uninjected WT and control MO-injected embryos exhibited similar gross morphology of TG neurons and their projections. However, Mecp2 morphants showed significantly less peripheral projections from TG (**Figure 1E**).

### Mecp2 Knockdown Decreases Projections from Sensory Neurons

**FIGURE 2:**
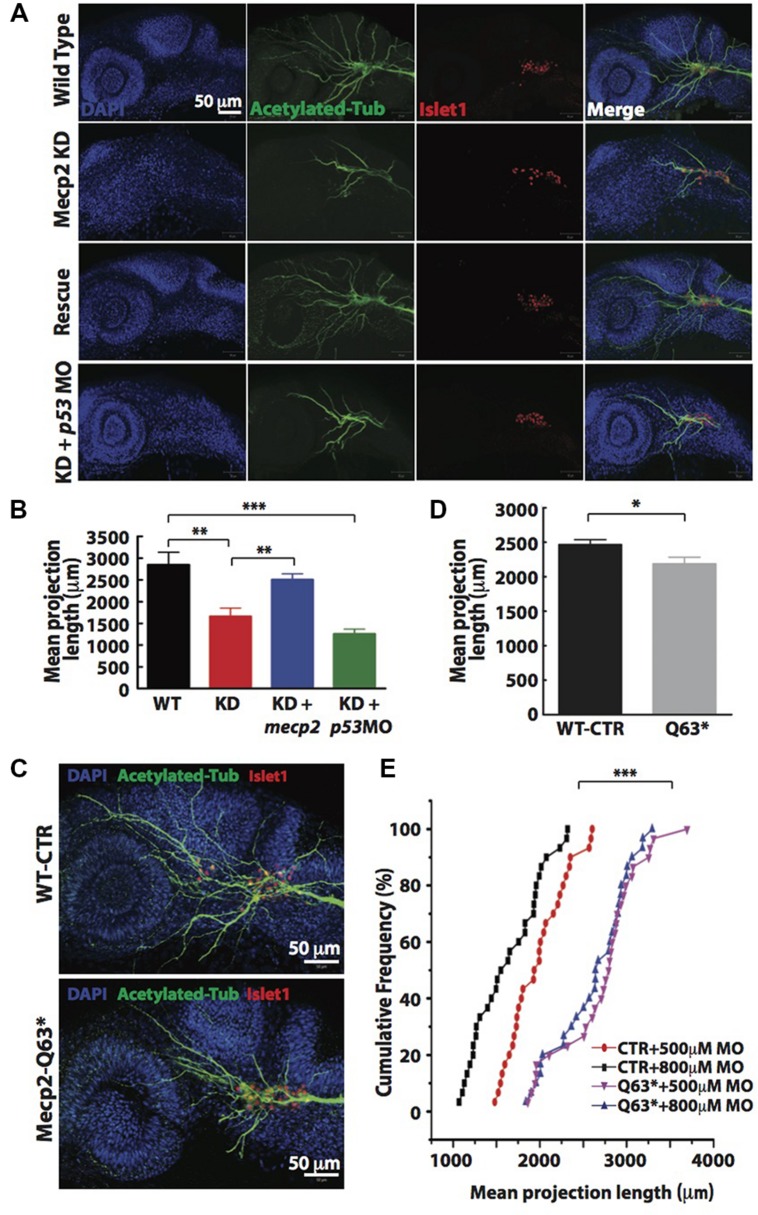
**Knockdown of Mecp2 affects the axonal projection of trigeminal ganglion (TG) cells.** Structure of peripheral dendritic projection and nerve pathways revealed by axon marker, acetylated tubulin (green), a general nucleus marker, DAPI (blue) and a neuronal marker, islet1 (red) at 24 hpf **(A)**. Representative images showing peripheral axonal projections from sensory neurons of TG in wild-type controls (WT, top panel), Mecp2 morphants (second panel from top), Mecp2 morphants rescued by coinjecting mRNA of Mecp2 (third panel from top) and Mecp2 morphants coinjected with *p53* MO (lower panel) **(A)**. Quantitative analysis of total peripheral projections of trigeminal sensory neurons, with graph presenting total length of peripheral projections from trigeminal ganglia per zebrafish **(B)** *N* > 10. Representative images showing peripheral axonal projections from sensory neurons of TG in wild-type controls (WT, top panel) and *mecp2^Q63^∗^/Q63^∗^^* mutants (Mecp2-Q63, bottom panel) **(C)**. Graph showing quantitative analysis of peripheral projections (total length) from trigeminal ganglia per zebrafish in wild-type controls and *mecp2^Q63^∗^/Q63^∗^^* mutants **(D)**. Graph showing total neurite length of controls (CTR) and *mecp2^Q63^∗^/Q63^∗^^* mutants (Q63) injected with either 500 or 800 μM of *mecp2* MO **(E)**. Scale bar = 50 μm. Each individual dot on graph **(E)** represents individual embryos. *N* = 30 in each group. (^∗^*P* < 0.05, ^∗∗^*P* < 0.01, ^∗∗∗^*P* < 0.0001, student *t*-test for **(D)** and one-way ANOVA with Bonferroni’s *post hoc* test for **(B,E)**].

Visibly less arborizations of peripheral projections from TG were observed in Mecp2 morphants (**Figure 1E**). Thus, we next quantify this defect by measuring the total projection length in each embryo (as mean projection length in μm). Knocking down of Mecp2 indeed inhibits growth and branching of the peripheral axons, resulting on average a 45% decrease in total length of peripheral axon projections as compared to controls. A potential caveat in interpreting these results is that some MOs can activate p53, thus causing non-specific neural defects (Robu et al., 2007; Eisen and Smith, 2008). We therefore routinely used *p53* MO to relieve possible non-specific neural cell death caused by toxicity effects of MOs (Robu et al., 2007). We found that embryos injected with *mecp2* MO together with *p53* MO showed similar phenotypes in sensory neurons compared to those injected with *mecp2* MO alone. To further ensure that these phenotypes are not due to off-target effects, we performed rescue experiments by co-injection with *mecp2* mRNA (**Figures 2A,B**). We found phenotypes of Mecp2 morphants could be reversed by co-injection with *mecp2* mRNA (**Figures 2A,B**). These results suggest that Mecp2 plays a key role in axonal development in zebrafish sensory neurons.

To further verify the specificity of these morpholino-mediated phenotypes, we also examined neurite projections in *mecp2*-null mutants (Q63^∗^). These *mecp2*-null fish exhibit clear altered behavioral alterations such as spontaneous and sensory-evoked motor anomalies, as well as defective thigmotaxis and have a relatively shorter lifespan than WT fish (Pietri et al., 2013). These *mecp2*-null embryos also show defect in TG projections as compared to WT control embryos, with about 15–20% decrease in the mean dendrite length of TG neurons as compared to WT control embryos (**Figures 2C,D**). Since this *mecp2*-null mutant is generated from a mutation that putatively truncated the protein at amino acid position 63, the truncated Mecp2 protein may still have residual Mecp2 activity. Thus, we next carried out titration experiments by injecting two different doses (500 and 800 μM) of *mecp2* MO in WT-CTR or Q63^∗^ mutant embryos (**Figure 2E**). This is to further rule out any off-target effects from *mecp2* MO and to determine if this truncated Mecp2 protein in *mecp2*-null embryos is still functional in promoting TG projections. WT-CTR embryos showed more severe defects in the mean projection length upon injection with higher concentration of Mecp2 MO. However, the increasing concentrations of *mecp2* MO did not cause further projection defects in Q63^∗^ mutants (**Figure 2E**), suggesting that there is no residual Mecp2 activity in *mecp2*-null mutant embryos, at least in these sensory neuron projections at the time point examined. These data obtained by titrating the Mecp2 MO in a null genetic background as compared to control embryos, further confirmed the specificity of our *mecp2* MO.

### Cell Autonomous Effect of Mecp2 on Sensory Neurons During Embryonic Development

**FIGURE 3:**
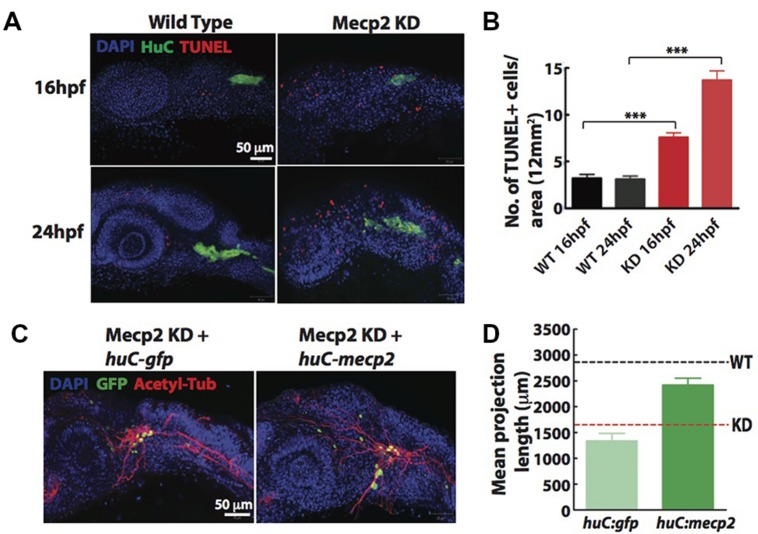
**Cell autonomous effect of Mecp2 on sensory neurons during embryonic development.** Embryos were injected with *mecp2* MO and labeled with TUNEL. Antibody against HuC was used to label cell bodies in TG. Representative images showing TUNEL labeling in TG in wild-type controls (WT, left panel), Mecp2 morphants (right panel) (Blue – DAPI, Green – HuC and Red – TUNEL) **(A)**. Quantitative analysis of TUNEL-labeled cells (indicative of apoptosis) at 16 and 24 hpf were presented in graphs showing total number of TUNEL positive cells per 12 mm × 12 mm in the head **(B)**. Representative images showing peripheral axonal projections from sensory neurons of TG in Mecp2 morphants rescued by coinjection with *huC-gfp* control plasmids (left panel) or *huC-mecp2* plasmids (right panel) **(C)**. Quantitative analysis of total peripheral projections of trigeminal sensory neurons, with graphs presenting total length of peripheral projections from trigeminal ganglia per zebrafish **(D)**. *N* > 10 in each group. Scale bar = 50 μm (^∗∗∗^*P* < 0.0001, one-way ANOVA with Bonferroni’s *post hoc* test).

Since a high rate of transient apoptosis during neurodevelopment is crucial for normal neurodevelopment in zebrafish (Cole and Ross, 2001), we examined the morphants using terminal deoxynucleotidyl transferase (TdT)-mediated dUTP nick-end labeling (TUNEL) labeling at 16 and 24 hpf. Knocking down Mecp2 increases the level of apoptosis at 16 and 24 hpf as compared to control (**Figures 3A,B**). There were an average of 7.5 and 14.0 TUNEL-positive cells per area of 12 mm × 12 mm in the head of morphants at 16 and 24 hpf respectively, as compared to the 2.0–4.0 TUNEL-positive cells in control embryos at both time points (**Figures 3A,B**). However, very few of the TG neurons were TUNEL-positive as shown by a low number of TUNEL-positive cells colocalized with a pan neuronal marker, HuC that labels differentiating neural cells (**Figure 3A**). The expression pattern of HuC in the early differentiating neurons was previously described (Park et al., 2000; Sato et al., 2006). HuC highlighted the first differentiated trigeminal neurons at 11 hpf. By 24 hpf, the TG has an average of 37 ± 3 neurons, and all these neurons express HuC. Other proteins such as Islet1, Trpa1b and P2x3b are also expressed in the TG, but are only expressed in different subpopulations of the trigeminal sensory neurons (Caron et al., 2008). Therefore, HuC was used as a marker to identify whether specific genes of interest are localized in all or only a subset of trigeminal sensory neurons (Pan et al., 2012). These studies indicated HuC as an ideal promoter to identify neurons in the TG.

To rule out non-cell-autonomous effect on sensory neurons from this general increase in apoptosis of the surrounding cells, we generated a construct with HuC promoter driving the expression of Mecp2 in neurons only. Since GFP intensity varies in different cells and low GFP intensity in most projections does not allow proper and unbiased tracings, we quantified total length and number of projections using acetylated tubulin as a marker (**Figures 3C,D**). Expression of *huC-mecp2* in Mecp2 morphants was able to rescue reduction in axonal projections in TG neurons (**Figures 3C,D**). The rescue is not as efficient as compared to that of *mecp2* mRNA, because not all TG cells express Mecp2 under the HuC promoter. Nonetheless, the rescue by *huC-mecp2* is significant and *huC-gfp* control does not have any effect on axonal projections in Mecp2 morphants. Taken together, these results confirm that the morphant phenotype is not caused by an increase in apoptotic cells in the vicinity of TG. Our data suggest that defects in sensory neurons resulting from Mecp2 knockdown represent a cell-autonomous effect.

### Mecp2 Regulates Transcriptional Activation of Axon Guidance Cues

**FIGURE 4:**
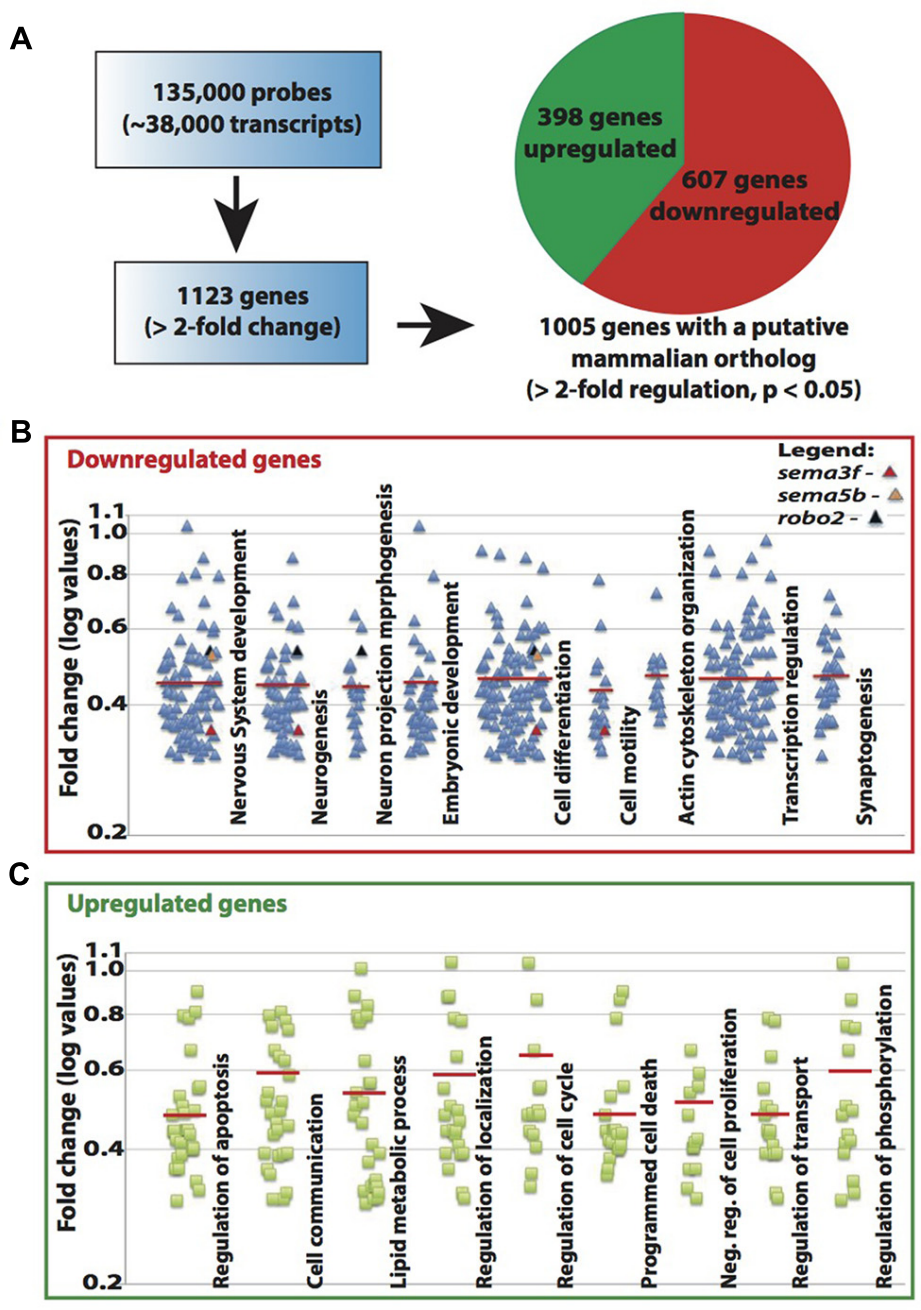
**Mecp2 morphants showed decreased expression of axon guidance cues.** Schematic diagram showing the number of genes that are upregulated or downregulated in Mecp2 deficient embryos **(A)**. Out of the 1005 genes that were found to have putative mammalian orthologs, 607 were downregulated and 398 were upregulated (minimum twofold change; significance was established at *p* < 0.05) in Mecp2 morphants **(A)**. Dot plots showing downregulated **(B)** and upregulated **(C)** genes. Each dot represent a single gene with at least twofold increased or decreased in expression presented as log values (base10) on *y*-axis. Relative fold change of downregulated genes, *sema3f* (0.352568), *sema5b* (0.523226) and *robo2* (0.537693) were highlighted as colored dots (red, orange, and black respectively) in their respective functional groups.

The known function of MeCP2 mainly involves its binding to methylated-CpG islands as well as activating and suppressing transcription. We therefore sought to examine the role of Mecp2 in regulating gene expression in zebrafish. Expression profiling was undertaken using a 135K-zebrafish gene expression array (∼38,000 known transcripts), comparing the transcriptome of wild-type controls with Mecp2 morphants. A total of 1123 genes had a minimum of 2-fold change over control (*p* < 0.05), but only 1005 of which had a mammalian (human or mouse) ortholog (http://www.ncbi.nlm.nih.gov/geo/ – accession number GSE71173). Out of these 1005 genes, 607 were down-regulated and 398 were up-regulated (minimum twofold change; significance was established at *p* < 0.05) in Mecp2 morphants, and 118 genes (43 down-regulated and 75 up-regulated) are without human orthologs (**Figure 4A**). These results strongly suggest that knocking down Mecp2 could either activate or repress transcription. Functional analysis of these 1123 genes *in silico* using the program Database for Annotation, Visualization and Integrated Discovery (DAVID) yield the following. Mecp2-regulated genes in zebrafish appear to function in diverse cellular processes (**Figures 4B,C**). Many of the up-regulated genes had roles in proliferation, cell communication and metabolism. By contrast, many of the down-regulated genes had functions related to neuronal development, including neurogenesis, neuronal projection morphogenesis, cell migration and synaptogenesis (**Figures 4B,C**). Since Mecp2 knockdown resulted in projection defects in sensory neurons, we searched for genes with axon guidance functions in our microarray that can potentially be involved in Mecp2-mediated effects. *sema3f*, *sema5b* and *robo2* were found in functional clusters involved in mammalian brain and nervous system development, specifically in neuronal projection morphogenesis, and they were strongly down-regulated by knocking down Mecp2.

**FIGURE 5:**
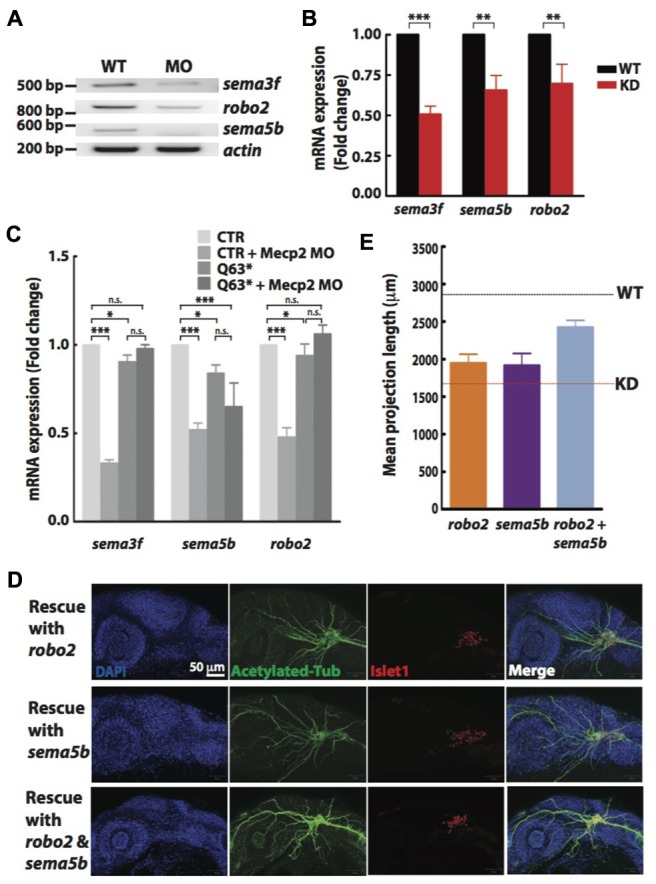
**Sema5b and Robo2 rescue Mecp2-induced defects in axonal projections in TG.** Representative gel images showing *sema3f*, *sema5b* and *robo2* mRNA levels regulated by Mecp2 **(A)**. Graph presenting quantitative RT-PCR of the three above mentioned genes in WT or Mecp2 MO injected embryos **(B)**. Graph showing expression of *sema3f*, *sema5b* and *robo2* mRNA in CTR and *mecp2^Q63^∗^/Q63^∗^^* mutants using RT-PCR **(C)**. Data are presented as fold expression of each gene normalized with housekeeping gene, *beta-actin* to value 1. (^∗^*P* < 0.05, ^∗∗^*P* < 0.01, ^∗∗∗^*P* < 0.001, two-way ANOVA with Bonferroni’s *post hoc* test). Sema5b and Robo2 rescue axonal projection of TG cells. Representative images showing peripheral projections of trigeminal sensory neurons in embryos injected with *mecp2* MO together with mRNA of Sema5b, Robo2 or both **(D)**. Quantitative analysis of total peripheral projections from trigeminal sensory neurons, with graph presenting total length of peripheral projections from trigeminal ganglia per zebrafish **(E)** (Blue – DAPI, Green – acetylated tubulin, Red – islet1). *N* > 10 in each group, Scale bar = 50 μm.

Non-quantitative and quantitative RT-PCR validated that mRNA levels of *sema3f*, *sema5b* and *robo2* were indeed reduced in Mecp2 morphants (**Figures 5A,B**). Our results suggest that Mecp2 plays an important role in the expression of these axon guidance cues. To determine if these genes are also downregulated in *mecp2*-null mutants, we examined mRNA expression of *sema3f*, *sema5b* and *robo2* in control and *mecp2*-null embryos (**Figure 5C**). There are modest but significant changes in *sema3f*, *sema5b* and *robo2* mRNA expression level in *mecp2*-null mutant as compared to WT control embryos. The modest change in the mRNA expression in *mecp2*-null mutants likely contributed to the subtle defects in TG projections in *mecp2*-null mutants. Whereas the bigger change in expression of these axon guidance cues in *mecp2* MO-mediated knockdown embryos contributed to the more severe defects. Moreover, injection of *mecp2* MO into *mecp2*-null embryos did not affect mRNA levels of *sema3f*, *sema5b* or *robo2*. These results confirmed that there are possibly compensatory effects in *mecp2*-null mutants as compared to *mecp2* MO injected embryos.

### Sema5b and Robo2 are Essential for Mecp2-Mediated Defects of Axon Projections

Although there are changes in expression levels of class 3 semaphorins and their receptors in *Mecp2*-null mice, neither Sema3A or 3F has effects on repulsion or neurite outgrowth response of *Mecp2*-null olfactory axons (Degano et al., 2009). We also did not observe significant defects in embryos injected with morpholino targeting *sema3f* (Data not shown). Therefore, to further establish Robo2 and Sema5b-mediated Mecp2-dependent neuronal projection morphogenesis, we asked if the defects seen in Mecp2 morphants could be rescued by Robo2 and Sema5b. We co-injected mRNAs of *robo2* or *sema5b* or both together with *mecp2* MO. Our data showed that excess Robo2 or Sema5b alone modestly rescued defects observed in Mecp2 morphants (average of 70 and 67% of control, respectively as compared to the 50% in Mecp2 KD group) (**Figures 5D,E**). Importantly, coexpression of both Robo2 and Sema5b in Mecp2 morphants showed more projections from TG neurons (average of 90% of control) as compared to single gene rescue (**Figures 5D,E**). These results suggest that Robo2 and Sema5b expression plays a role in the neurodevelopmental phenotype associated with loss of Mecp2.

### Mecp2 Directly Regulates Transcription of *sema5b* and *robo2*

**FIGURE 6:**
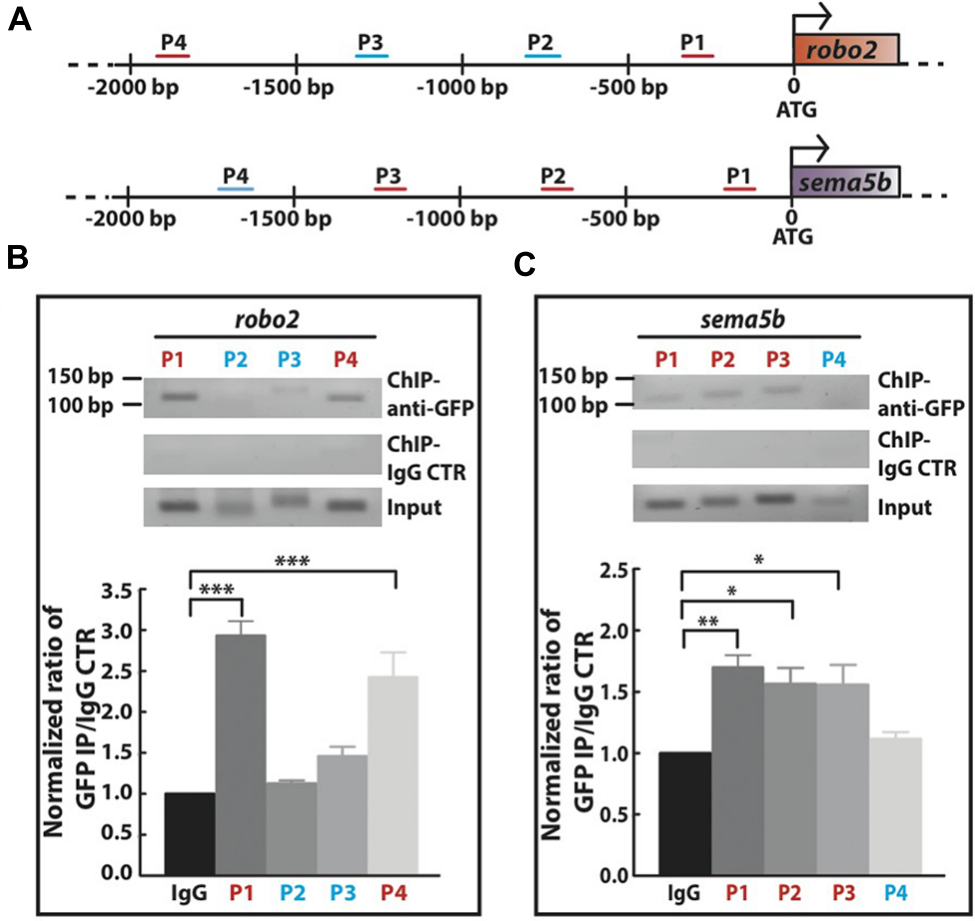
**Mecp2 directly regulates transcription of *robo2* and *sema5b* genes.** Chromatin immunoprecipitation (ChIP) analysis shows the recruitment of Mecp2 to *robo2* and s*ema5b* promoters. Schematic diagram of the 5′ promoter region of *robo2* and *sema5b*. Short horizontal lines (red and blue) labeled P1-4 indicate the relative positions of PCR products from the respective sets of primers on both promoter regions **(A)**. Representative gel images (top panel – anti-GFP pulldown, middle panel – IgG control pulldown and bottom panel – input) and quantitative analysis of ChIP assay performed on 24 hpf wild-type embryos overexpressing Mecp2 protein (GFP-tagged Mecp2) **(B,C)**. Anti-GFP antibody pulled down specific *robo2* and *sema5b* promoter regions [strong binding as indicated by the PCR products (amplify by their respective primers) in red, and very weak or no binding as indicated in blue]. Graphs show fold difference ratio of ChIP with anti-GFP over ChIP with IgG control normalized to value 1 **(B,C)**. (^∗^*P* < 0.05, ^∗∗^*P* < 0.01, ^∗∗∗^*P* < 0.001, two-way ANOVA with Bonferroni’s *post hoc* test).

Next, we investigated whether Mecp2 directly regulates Robo2 and Sema5b expression by interacting with their promoters. We carried out conventional chromatin immunoprecipitation (ChIP) assay on 24 hpf wild-type embryos injected with *mecp2-gfp* using GFP antibody, followed by site-specific PCR analysis. Primers were designed within 2000 bp region upstream of translational start site of both *sema5b* and *robo2* promoters. Our results showed that specific regions on the promoters of these two mentioned genes co-immunoprecipitated with Mecp2 *in vivo*, suggesting that their transcription is directly subjected to Mecp2 regulation (**Figure 6A**). Quantification of PCR products showed weak binding of Mecp2 to the promoter of *sema5b* at P4 (within –1824 to -1723) and relatively strong binding at P1, P2, and P3 (within -155 to -57, -792 to -681, and -1310 to -1177) regions (**Figures 6A,C**). On the other hand, Mecp2 binds strongly to *robo2* promoter at P1 and P4 regions (within -349 to -222 and -2045 to -1916), and does not bind or only binds very weakly to P2 and P3 regions (-849 to -730 and -1374 to -1236) (**Figures 6A,B**). These results suggest that Mecp2 is likely to regulate transcription of Robo2 and Sema5b, through its association with the promoters of both genes.

### Mecp2-Mediated Defects of Axon Projections Leads to Diminished Sensory Response

**FIGURE 7:**
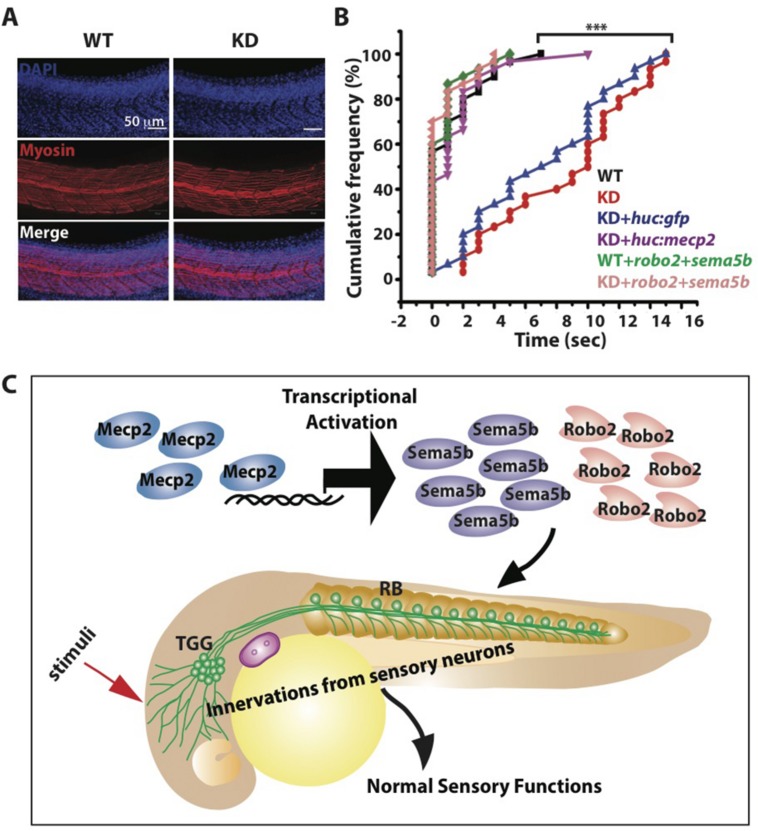
**Mecp2-mediated defects of axon projections leads to diminished sensory response.** Representative images showing arrays of muscle fibers in WT control and Mecp2 morphants (KD) embryos immunostained with monoclonal antibody A4.1025 (myosin) **(A)**. In all panels, anterior is to the left. (Blue-DAPI, red-myosin) Scale bar = 50 μm. Graphs showing rates of response upon tactile stimuli (red arrow in C) from the six experimental groups of WT and KD, including WT and KD coinjected with various rescue constructs indicated on the graph, upon tactile stimuli applied to the same region specified **(B,C)**. Proposed model shows Mecp2 plays a part in primary sensory projections through regulating transcription of Sema5b and Robo2 **(C)**. Each individual dot **(B)** represents individual embryos. *N* > 30. (^∗∗∗^*P* < 0.0001, one-way ANOVA with Bonferroni’ *post hoc* test).

Innervation of sensory neurons is essential for sensory functions, which can be assessed by the ability of the embryo to respond to tactile stimuli (Sagasti et al., 2005; Carmean and Ribera, 2010; Low et al., 2011). We found normal muscle striations in Mecp2 deficient embryos (**Figure 7A**), but these embryos are less responsive to tactile stimuli as compared to controls at 48 h after *mecp2* MO injection (**Figures 7B,C**). WT control embryos responded to head tactile stimuli within an average of approximately 0.5 s upon tactile stimulation, whereas Mecp2 morphants took an average of 10.0 s to respond.

Importantly, HuC promoter specific expression of Mecp2 in TG neurons rescued the diminished sensory response in Mecp2 morphants, reaffirming that the sensory behavioral phenotype is a cell autonomous effect resulting from a loss of Mecp2 (**Figure 7B**). This diminished sensory response in Mecp2 morphants mirrors the sensory deficit phenotypes observed in Rett patients, mouse models (Devarakonda et al., 2009; Downs et al., 2010; Battaglia, 2011; Samaco et al., 2013) and zebrafish *mecp2*-null mutant (Pietri et al., 2013). To determine if Robo2 and Sema5b directly play a role in Mecp2–mediated sensory response, we coexpressed Robo2 and Sema5b in Mecp2 morphants (**Figure 7B**). This led to restoration of sensory responses in Mecp2 morphants to levels comparable to WT controls, indicating that Robo2 and Sema5b are downstream proteins of Mecp2 that are responsible for mediating MeCP2-dependent sensory responses (**Figure 7C**).

Taken together, our results show that Mecp2 plays a role in embryonic development of zebrafish. Mecp2 could function by regulating the expression of genes acting during formation of peripheral neurites by sensory TG neurons, which is essential for sensory response (working model illustrated in **Figure 7C**).

## Discussion

Human males with mutations in *MECP2* gene normally do not survive, whereas females showed normal postnatal development till 6–18 months of age, when typical neurological symptoms started to appear (Amir et al., 1999; Armstrong, 2005). Male rhesus and cynomolgous fetuses carrying *MECP2* mutations were miscarried, while females did not show any obvious phenotypes at least for the first 4 months after birth during the study period (Liu et al., 2014). The human disease manifestations were profoundly different from those seen in mouse Rett syndrome models where all animals survived, and males but not females displayed Rett syndrome phenotypes at an early age (Guy et al., 2011). Despite showing clear behavioral alterations during early development, *mecp2^Q63^∗^^* mutant fish also survived but has relatively shorter lifespan than WT fish (Pietri et al., 2013). In addition, this *mecp2^Q63^∗^^* mutant exhibits motor defects compatible with motor phenotypes observed in Mecp2-null mouse models and Rett syndrome patients. This *mecp2^Q63^∗^^* mutant fish also showed delayed response to tactile stimulations (Pietri et al., 2013) which is in agreement with defects exhibited by Mecp2 morphants and MeCP2 deficiency in mice. Partial MeCP2 deficiency in mice resulted in reduced pain recognition in a hot plate assay (Samaco et al., 2008). Mecp2 knockdown in zebrafish resulted in retarded responses to tactile stimuli while exhibiting normal muscle striations as compared to controls indicating that Mecp2-knockdown-induced defect in sensory neuron projections is indeed responsible for the diminished touch sensitivity observed.

The presumably null *mecp2^Q63^∗^^* mutation is a nonsense mutation generated using ENU mutagenesis approach, putatively truncated the protein at position 63 and does not correspond to any known human *MECP2* mutations. In mice, the two *Mecp2* splice variants (*Mecp2-e1* and *Mecp2-e*2) differing only in the first 26 amino acids, show dramatically different functions such that forced expression of MeCP2-e2 but not MeCP2-e1 promotes cell death (Dastidar et al., 2012). Although the non-lethality of this mutant fish could be that the truncated protein still had functions essential for sensory neuronal projections in zebrafish, our data from titration experiments using different concentrations of *mecp2* MO rule out this possibility. Injection of *mecp2* MO into WT-CTR but not these *mecp2^Q63^∗^^* mutants affected innervations of TG neurons and expression of the axon guidance cues examined, confirming specificity of our *mecp2* MO. Despite the absence of functional Mecp2, the expression of axon guidance cues such as Sema5b and Robo2, are only modestly but significantly decreased in these *mecp2^Q63^∗^^* mutants as compared to Mecp2 morphants. Thus, explaining the milder TG projection phenotypes observed in *mecp2^Q63^∗^^* mutants as compared to Mecp2 morphants and also suggesting possible compensatory effects in *mecp2^Q63^∗^^* mutants. Possible compensatory mechanisms include the presence of another genetic alteration in the *mecp2*-null mutants that can suppress effects of the *mecp2^Q63^∗^^* mutation, functional compensation through other proteins that have MBD and TRD domains or Mecp2-independent positive feedback loop for regulating Mecp2 target genes (Pietri et al., 2013). In fact, it has recently been shown that genetic compensation in zebrafish mutants but not morphants is the key reason underlying the difference in phenotypes between mutants and knockdowns (Rossi et al., 2015).

Zebrafish and human MeCP2 proteins share only about 43% amino acid sequence identity (and 46.3% identity with *Xenopus*), but the conserved domains are apparent (Coverdale et al., 2004) and Mecp2 may thus serve similar functions in mammals and fish. Zebrafish Mecp2 protein is more similar to the mammalian MeCP2α isoform than the MeCP2β in terms of N-terminus and genomic organization (Coverdale et al., 2004). Studies with mutant Mecp2 in *Xenopus* showed deficits in binding of Mecp2 to a corepressor complex (Stancheva et al., 2003). Mecp2 knockdown in zebrafish reduces metabotropic-type glutamate receptor 2 (mGluR2), which was also observed in Rett syndrome patients (Blue et al., 1999). Some of the other Mecp2-regulated genes that we found in zebrafish, such as *atxn1* and *pax6*, were previously implicated in developmental delays in humans (Kim et al., 2011; Celestino-Soper et al., 2012). Therefore, MeCP2-regulated genes found in mammalians are likely to be involved in similar processes in zebrafish.

Functional categorization of all the Mecp2-regulated genes on our array suggest that Mecp2 is involved in transcriptional down-regulation of genes with roles in general cell maintenance, but up-regulation of genes with roles in neuronal development, specifically in neuronal differentiation, axon projections and synaptogenesis. One possible explanation for MeCP2 acting as transcription activator or repressor, is the binding of MeCP2 to either 5-hydroxymethylcytosine (5hmC) or 5-methylcytosine (5mC) as shown in a recent study using mice. MeCP2 binding to 5hmC can facilitate transcription in neural cell types, but represses transcription when bound to 5mC containing DNA (Mellén et al., 2012). However, the 5hmC and 5mC content in the same genes vary between different cell types. These variations can therefore explain, or at least in part, for the very few overlaps in MeCP2-dependent regulated genes found from different studies, especially from studies using different cells and different parts of the mammalian brains (Guy et al., 2011). For example, one of the most characterized target gene of MeCP2 – *BDNF*, does not consistently appear to be regulated by MeCP2 in all the arrays (Guy et al., 2011). Ratios of 5hmC/5mC in mouse *Sema5b* and *Robo2* (the two genes that we found transcriptionally activated by Mecp2 in zebrafish) are approximately 1 or much higher than 1 (ratio of 3) in purkinje cells (PCs) and specialized Bergmann glial (BG) cell population, but is only about 0.2–0.4 in granule cells (GCs) (Mellén et al., 2012). Therefore, suggesting that MeCP2 probably activates transcription of both *Sema5b* and *Robo2* in PC and BG but represses transcription of both genes in GC cells.

Our results show that Mecp2 knockdown causes defects in projections from sensory neurons through activating transcription of axon guidance cues represented by Sema5b and Robo2. These defects in zebrafish emulate sensory and neuromotor deficits seen in Rett syndrome patients (Samaco et al., 2008; Devarakonda et al., 2009; Downs et al., 2010; Battaglia, 2011). Although Rett syndrome patients have sensory deficits, this aspect of the disorder is still largely uncharacterized. *sema3f, sema5b* and *robo2* are genes that have roles in axon projections and synaptogenesis and were significantly down-regulated in Mecp2 morphants (**Figures 4** and **5**). These known axon repelling guidance cues appear to have axon promoting functions in sensory neurons in zebrafish (at least for both Sema5b and Robo2). Previous studies in mice showed guidance cues such as Slits (ligand for Robo receptor) that typically act as axon repellents may also act to attract some axons (such as DRG, trigeminal sensory neurons and retinal axons inside the optic fiber layer) (Wang et al., 1999; Nguyen Ba-Charvet et al., 2001; Jin et al., 2003; Ma and Tessier-Lavigne, 2007). Semaphorins typically cause repulsion and collapse of axons but could on the other hand promote dendrite growth in hippocampal neurons (Ng et al., 2013). Therefore, it could be possible that semaphorins can also promote growth of some axons as observed for Slit/Robo. Semaphorins are widely expressed in the developing nervous system of mice, but there are no known roles of Sema5b specifically. Previous studies in mouse models demonstrated that semaphorins act as guidance cues for axon targeting, cell migration, cell death or synapse formation during the development of nervous system (Koncina et al., 2007). Severe abnormality in peripheral nerve projections, which includes trigeminal, facial, vagus, accessory and glossopharyngeal nerves were observed in Sema3D knockout mouse (Taniguchi et al., 1997).

The Robo2 protein is highly conserved from fly to humans, and is known to function in developing nervous systems with roles in axon guidance, synaptogenesis and cell migration (Yeo et al., 2004; Campbell et al., 2007; López-Bendito et al., 2007; Andrews et al., 2008; Pan et al., 2012). Mice deficient in Robo2 demonstrated obvious axon guidance errors, with a more prominent effect on mice deficient in both Robo1 and Robo2 (López-Bendito et al., 2007). Like Robo2, Sema5b is also a ligand-binding receptor. Besides its role as a guidance cue, little else is known about its role in neuronal development. Interestingly, Robo2 or Sema5b can both modestly rescue defective projections from TG neurons in Mecp2 morphants. Although there are no known expression studies on Sema5b in zebrafish, Robo2 was found to be expressed in a subpopulation of HuC positive cells of the TG in zebrafish embryos at 24 hpf (Pan et al., 2012). Therefore, partial rescue of the phenotype observed upon expressing Robo2 alone could be because not all TG neurons express Robo2. It is also possible that both Robo2 and Sema5b are working together and are not able to achieve significant rescue effect as an individual. Concurrent expression of both Robo2 and Sema5b can rescue this defect to approximately control levels, suggesting that Robo2 and Sema5b plays important role in mediating the effects of Mecp2 in regulating projections of sensory neurons. In contrast, although we found a decrease of Sema3f expression in Mecp2 morphants, this decrease did not translate to significant defects, at least in the gross morphology of zebrafish embryos and also in projections of sensory neurons, which is likely due to compensatory effects from other class 3 semaphorins. Similar observation on mice was reported, where abnormal levels of class 3 semaphorins and their receptors were also observed in the olfactory system in *Mecp2*-null mice. But neither Sema3A nor 3F elicited any difference in repulsion or neurite outgrowth response of *Mecp2*-null olfactory axons (Degano et al., 2009).

In summary, our data using *mecp2*-null mutants and *mecp2* MO-mediated knockdown approach show that Mecp2 is important for peripheral innervation of sensory neurons in the zebrafish. Mecp2 knockdown in zebrafish model recapitulates sensory deficits of Rett syndrome in humans. We also found expression of well-known axon guidance cues such as Sema5b and Robo2 positively regulated by Mecp2 through its interactions with specific regions on both promoters. These guidance cues are essential for the Mecp2-dependent innervations from sensory neurons and sensory response during embryonic development.

## Materials and Methods

### Injection of Zebrafish

Wild-type zebrafish of the AB strain were maintained under standard conditions of fish husbandry. Freshly fertilized zebrafish eggs were injected with mRNA (100 ng/μl), plasmid DNA (40 ng/μl), *mecp2* morpholino (800 μM), *robo2* morpholino (200 μM) and *sema5b* morpholino (500 μM) at the one- to two-cell stage in a volume of approximately 1 nl. Approximately 200 embryos were injected for each morpholinos or overexpresson constructs. At least 30 embryos were analyzed for each experimental group used per experiment. For experiments using *mecp2*-null zebrafish embryos, *mecp2* splice blocking morpholino was also injected into *mecp2^Q63^∗^/Q63^∗^^* mutant (*mecp2*-null) and its wild-type control embryos (WT-CTR) (both in the Nacre background). The injected embryos were cultured at 28°C, and embryos were fixed at specific developmental stages for further analysis. Morpholinos were purchased from GeneTools. Splice-blocking morpholino (5′-CTCACCTCTGCTGACAACAAAATAA-3′) was selected for knocking down Mecp2. This splice-blocking MO that allows efficiency of MO to be determined through PCR, was used for all the Mecp2 morphants shown here. The control morpholino (5′-CCTCTTACCTCAGTTACAATTTATA-3′) and *p53* morpholino (5′-GCGCCATTGCTTTGCAAGAATTG-3′) used were the scrambled sequence and a translational blocker respectively from Gene Tools. All zebrafish experiments were in compliance and approved by the Singapore National Advisory Committee on Laboratory Animal Research.

### Cloning of Zebrafish *mecp2*, *sema5b*, and *robo2*

cDNAs were synthesized from 24 hpf wild-type embryos using cDNA synthesis kit (Invitrogen). These cDNA templates were used for PCR amplification of the full-length zebrafish *mecp2*, *sema5b* and *robo2*. The PCR products were cloned into TOPO vector and subsequently sub-cloned into either pCS2-GFPxlt or pSP64T expression vector for over expression studies and rescue experiment. Full-length zebrafish *mecp2* was cloned into *huC* promoter plasmid to induce specific expression in subsets of TG neurons. GFP-expressing embryos were screened by fluorescent microscopy to determine for expression of injected constructs. Only embryos that expressed GFP were used for all downstream experiments and analysis.

### Whole-Mount *In Situ* Hybridization

Purified plasmid was linearized by selected restriction enzymes and used as templates for *in vitro* transcription using T7 or SP6 RNA polymerase to generate Digoxigenin (DIG)-labeled sense and antisense probes using DIG RNA labeling kit (Roche). Whole-mount *in situ* hybridizations were performed following routine protocols.

### Antibody Staining and Terminal Deoxynucleotidyl Transferase (TdT)-Mediated dUTP Nick-End Labeling (TUNEL) Assay

Whole-mount antibody staining on zebrafish embryos was performed according to standard protocols. The following antibodies were used: rabbit anti-GFP (1:1000; Abcam), monoclonal anti-acetylated tubulin (1:500; Sigma), monoclonal anti-islet [1:100; The Developmental Studies Hybridoma Bank (DSHB)], monoclonal antibody A4.1025 (skeletal muscle myosin) (1:20; DSHB) and mouse anti-HuC/D (1:1000; Invitrogen). For confocal microscopy, appropriate Alexa Fluor-conjugated secondary antibodies (1:500; Molecular Probes) were used for signal detection. Embryos were counterstained with 4,6-diamidino-2-phenylindole (DAPI) to visualize cell nuclei when required. For TUNEL assay, the embryos were made more permeable by treating with proteinase K and 0.1% sodium citrate prior to the standard protocol as recommended by the manufacturer (Roche).

### *In Vitro* Transcription of Capped mRNA

Plasmids encoding *mecp2*, *sema5b* and *robo2* were linearized, and capped, full-length mRNA was transcribed from this template using the mMessage mMachine Kit (Ambion). The mRNA was injected into one- to two-cell stage embryos either alone or in combination with a morpholino.

### Zebrafish Gene Expression Microarray

Three independent pools of 24 hpf zebrafish Mecp2 morphant embryos were compared to three independent pools of 24 hpf control embryos. RNA extraction was done using RNeasy mini kit (QIAGEN). The triplicate embryo pools made a total of six hybridizations. cDNA synthesis, cDNA labeling, microarray hybridization and washing were performed following the manufacturer’s instructions (Roche NimbleGen). The 135K NimbleGen array contains 135,000 probes targeting 38,489 transcripts derived from the Ensembl database built on the Zv7 assembly (37,157 zebrafish genes identified). Array hybridization was done using Maui Hybridization System and was scanned using Axon GenePix 4000B Scanner. Data extraction and image processing were performed using NimbleScan software v.2.6 and the Robust Multichip Average (RMA) algorithm used to generate gene expression values. The normalized data was subsequently analyzed using ArrayStar. Threshold was set at twofold change with a 95% confidence interval by student’s *t*-test with Benjamini Hochberg False Discovery Rate analysis.

### Quantitative Real-Time PCR

One microgram of total RNA was used as starting material. Reverse transcription was carried out with Invitrogen’s SuperScript III First-Strand Synthesis kit using oligo(dT) primers. Gene specific primers were used to amplify *sema3f* (fw-5′ CAACCAGTACTGCCAAGACTAC 3′; rv-5′ TCCTGGTGGTGTCTCCTATT 3′), *sema5b* (fw-5′ CTGCTGTCTTTCCTGGTGTATG 3′; rv-5′ GTGGTGTTGCCCTTGTAGTT 3′) and *robo2* (fw-5′ GACATACCTCCATCAGGGTTTAG 3′; rv-5′ GAAACTGCAGCAGGAGAAGA 3′) by qPCR on a Biorad CFX96 RT PCT using SYBR Select master mix (Invitrogen). A sample volume of 12 μl was used for all assays. All samples and standards were run in triplicates. Three biological replicates were performed for each set of data normalized using housekeeping gene *beta-actin*.

### Chromatin Immunoprecipitation (ChIP)

Wild-type embryos were injected at one-cell stage with GFP-tagged *mecp2* RNA. They were harvested manually at 24 hpf, then dechorionated, deyolked and immediately crosslinked to DNA by direct addition of formaldehyde at a final concentration of 1% for 10 min at room temperature. Embryo tissues were lysed and sonicated (Sonics Vibra-Cell) to produce DNA fragments that are between 300 and 800 bp. After sonication, ChIP assay was performed according to Millipore protocol (Magna ChIP G Tissue Kit). Protein-DNA complexes were immunoprecipitated overnight at 4°C in the presence of specific anti-GFP antibody (Abcam) or control rabbit IgG (Santa Cruz Biotechnology). DNA was purified and used directly for PCR of *sema5b* and *robo2* promoters. Primers were designed within 2000 bp region upstream of translational start site of *sema5b* promoter (Primer 1: fw-5′ CTCTGCTGATCGTAAAGGTGTAT 3′; rv-5′ GTTAAAGAGGACAGACGGAGTG 3′, Primer 2: fw-5′ TCCAGCTCAGTTCAGTTCAAG 3′; rv-5′ TTGCGATTTCCAACATATACATAGC 3′, Primer 3: fw-5′ GGGTTCAATTCCCTACAGTCTT 3′; rv-5′ GGATGGATGGATTGATGGTTAGA 3′, Primer 4: fw-5′ TGTTCCACTTCTGGTCAGTTT 3′; rv-5′ TCTGAGGAGACCCAGCTTTA 3′) or *robo2* promoter (Primer 1: fw-5′ GAGGATCGTAAGGTGCTTCTG 3′; rv-5′ ACACCATCTCCTACTGTGTTTG 3′, Primer 2: fw-5′ ACACTGACATATTGGTGGTCAT 3′; rv-5′ CAGCTTTCCGTGGTGTTTATTC 3′, Primer 3: fw-5′ CTTAGAGCTGACACTGGAAGATG 3′; rv-5′ TGGGCGAACGGATAGATAGA 3′, Primer 4: fw-5′ TGCTGACACACCGCTAATC 3′; rv-5′ GAGGAATGCAAGGACCAATTTC 3).

### Behavior Studies

Wild-type embryos injected with either control morpholino (800 μM), *mecp2* morpholino (800 μM), or in combination with rescue constructs (∼40 ng for DNA and 100 ng for mRNA) were allowed to develop to 48 hpf under normal growth conditions before scoring was done. These embryos were dechorionated manually and subjected to tactile stimuli at the head region. Manual scoring was done by taking down the time required by the embryo to produce a response upon stimulation applied with the tip of a mounting needle. Zero seconds was recorded for embryos that gave an immediate reaction to the tactile stimuli.

### Imaging, Analysis, and Figure Preparation

Stained embryos were dissected from their yolk and mounted in 70% glycerol. High-resolution images of embryos were captured using a Zeiss LSM 710 confocal microscope (Carl Zeiss Pte Ltd, Singapore). Low-resolution images were obtained using a Zeiss SteREO Discovery V8 stereoscope equipped with an Axiocam ICc3 color digital camera (Carl Zeiss Pte Ltd, Singapore). For analysis of the neurite structure of TG cells, three-dimensional (3-D) reconstructions of the processes of all labeled cells were made from Z-series stacks of confocal images. The projection images were semi-automatically traced with NIH ImageJ using the NeuronJ plugin. The total length of processes in each individual embryo were subsequently measured and analyzed. All data were scrambled and only decoded after analysis was done blinded. A total of >30 embryos from >3 individual experiments were analyzed per group. Gel quantification of DNA for ChIP PCR was done using NIH ImageJ. Statistical significance (*P* < 0.05) was assessed using student *t*-test, one-way or two-way ANOVA with Bonferroni’ *post hoc* test, after normalization checked using Shapiro–Wilk normality test. Figures were assembled using Adobe Illustrator CS4.

## Author Contributions

WL designed and performed all experiments and analyzed all data; ZL analyzed data for microarray; VK designed some experiments and made critical edits on the manuscript; TP contributed key materials for the experiments and made critical inputs to the manuscript; EG initiated and directed the entire study, designed experiments, analyzed data and wrote the manuscript.

## Conflict of Interest Statement

The authors declare that the research was conducted in the absence of any commercial or financial relationships that could be construed as a potential conflict of interest.
